# Sex differences between atherogenic index of plasma and α-klotho levels in middle-aged and older adults: NHANES 2007–2016

**DOI:** 10.3389/fendo.2025.1521415

**Published:** 2025-04-07

**Authors:** Cheng Zuo, Zi-Duo Shen, Yaojun Lu

**Affiliations:** ^1^ Department of Cardiology, Central Hospital of Baotou, Baotou, China; ^2^ Stroke Center, Department of Neurology, The First Hospital of Jilin University, Changchun, China

**Keywords:** atherogenic index of plasma, klotho, sex differences, NHANES, lipid

## Abstract

**Objective:**

*Klotho* is an anti-aging gene, and the α-klotho protein it encodes reportedly has cardiovascular protective effects. The atherogenic index of plasma (AIP) is a novel and comprehensive lipid index that correlates with atherosclerotic burden and is a critical risk factor for cardiovascular diseases. There are no studies examining the relationship between AIP and α-klotho; thus, we aimed to explore this potential association.

**Methods:**

Data were extracted from the National Health and Nutrition Examination Survey 2007–2016 database, and the relationship between AIP and serum α-klotho levels was analyzed using weighted multivariate linear regression.

**Results:**

After adjusting for risk factors, AIP showed a significant negative correlation with the logarithm of serum α-klotho levels in women. The trend analysis and smoothed curve fitting showed a nonlinear dose–response relationship. Threshold effect analysis showed a significant difference in the association between AIP and α-klotho before and after the AIP break point (0.434). Subgroup analyses demonstrated that the negative association of AIP with α-klotho was consistent across subgroups. However, the correlation between AIP and serum α-klotho in men was not significant.

**Discussion:**

Our study provides new evidence for sex differences in the association between AIP and serum α-klotho levels.

## 1 Introduction

Since Kuro et al. discovered the *klotho* gene in 1997 during a study on spontaneously hypertensive mice, numerous studies have investigated its relationship with health (1–3). As an anti-aging gene, *klotho* is strongly expressed in the epithelial cells of the renal distal tubules and the brain’s choroid plexus (4). It encodes a 135-kDa transmembrane protein, known as the klotho protein, also referred to as the α-klotho protein, which consists of 1,012 amino acids (5). The α-klotho protein exists in two molecular forms: membrane-bound and secreted. The membrane-bound form primarily promotes phosphate excretion in the kidneys and maintains calcium-phosphorus balance. In contrast, the secreted form acts as a hormone-like cytokine, participating in processes such as anti-inflammation, antioxidation, anti-apoptosis, anti-fibrosis, anti-tumor activity, ion channel regulation, and insulin resistance (1, 6, 7). Studies in both mice and humans have consistently demonstrated that low α-klotho expression correlates with premature aging phenotypes, including osteoporosis, atherosclerosis, cognitive deficits, and immune deficiencies. Conversely, overexpressing α-klotho can reverse the progression of these aging-related conditions and prolong lifespan (8–11). Therefore, understanding the factors associated with α-klotho protein levels is critical for health monitoring and prognostic assessment.

The atherogenic index of plasma (AIP) is a newly discovered biomarker associated with lipid metabolism (12). It reflects the balance between triglyceride (TG) and high-density lipoprotein cholesterol (HDL-C) levels in the blood and serves as a sensitive biomarker for atherosclerosis. Studies have shown that AIP is significantly correlated with cardiovascular events and can effectively predict cardiovascular disease risk (13, 14). Therefore, AIP not only holds high diagnostic value in clinical practice but also provides a useful reference for early prevention and intervention. Previously, low-density lipoprotein cholesterol (LDL-C) was considered the primary therapeutic target for dyslipidemia; however, some studies have shown that even when target LDL-C levels are achieved, patients still face a high residual cardiovascular risk (15, 16). Further studies found that small dense LDL-C (sdLDL-C) poses a more significant atherogenic risk than LDL-C (17). However, the complexity and high cost of sdLDL-C testing have limited its clinical application. Recently, it has been demonstrated that AIP can indirectly reflect LDL-C particle size, quantify the degree of lipid metabolism abnormalities, and outperform traditional lipid indices (18, 19). Therefore, studying AIP appears more relevant than focusing on LDL-C. Given the importance of AIP as a marker of atherosclerotic risk and the potential role of α-klotho in lipid metabolism and cardiovascular health, there may be an association between AIP and α-klotho levels. Studying this relationship could have significant clinical implications. In clinical practice, directly measuring plasma α-klotho levels require specialized equipment and techniques, which are complex and costly. In contrast, AIP is a routine, simple, and low-cost test that can be performed through conventional blood testing. If α-klotho levels could be estimated using AIP, it would provide a more convenient and economical screening method, particularly in resource-limited settings. To our knowledge, no studies have investigated the relationship between AIP and α-klotho. Therefore, we aimed to examine this relationship in middle-aged and older US populations using data from the National Health and Nutrition Examination Survey (NHANES) 2007–2016.

## 2 Materials and methods

### 2.1 Study population

NHANES is a cross-sectional study that focuses on the health and nutritional status of the US population. It employs a complex multistage probability sampling method that includes face-to-face interviews, physical exams, and laboratory tests for data collection. The survey received ethical approval from the Ethics Review Board of the National Center for Health Statistics, and all participants provided written informed consent. Additional information is available on the NHANES website (www.cdc.gov/nchs/nhanes.htm).

**Figure 1:**
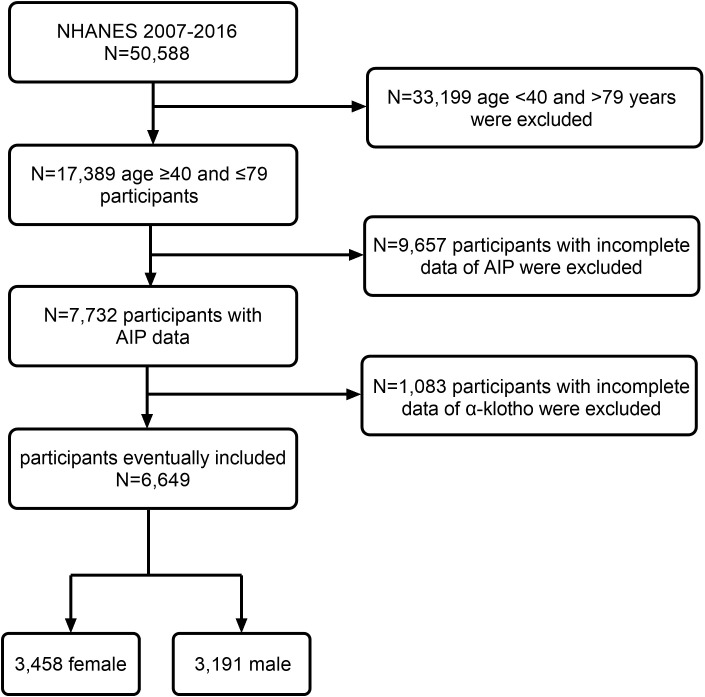
Flowchart of the study design and participants excluded from the study.

This study included 50,588 participants from a nationally representative sample of NHANES from 2007 to 2016. We excluded 33,199 participants aged <40 or >79 years. Among the participants, 9,657 had missing AIP data, and 1,083 had missing α-klotho data. Ultimately, this study included 6,649 participants comprising 3,458 women and 3,191 men. **Figure 1** shows the detailed screening process.

### 2.2 Assessment of AIP

A professional technician collected participants’ fasting TG and HDL-C levels. AIP was calculated using the formula:


$$\text{AIP}=\log_{10}TG/HDL-C$$

TG and HDL-C are expressed in mmol/L in the formula.

### 2.3 Assessment of serum α-klotho

Due to the lack of α-klotho data for individuals younger than 40 and older than 79, only participants aged 40–79 were included in this study. Notably, all original serum α-klotho samples were frozen, stored at -80°C, and sent to the University of Washington for analysis between 2019 and 2020. α-Klotho concentrations were determined using a commercially available enzyme-linked immunosorbent assay kit manufactured by IBL (Fujioka, Japan). Each sample was analyzed twice, and the average values were calculated. Comprehensive details regarding the laboratory methods and quality assurance protocols are available on the official NHANES website.

### 2.4 Assessment of covariates

On the basis of previous studies, we identified potential confounders for the relationship between AIP and serum α-klotho (20). These included age, race, education level, marital status, annual household income, physical activity, smoking behavior, drinking behavior, hypertension, diabetes, coronary heart disease, liver disease, kidney failure, and systemic immune-inflammatory index (SII). SII was calculated as follows:


$$\text{SII}=\frac{\text{platelet count}\left(\times 103\text{ cells}/\text{μL}\right)\times \text{neutrophil count}\left(\times 103\text{ cells}/\text{μL}\right)}{\text{lymphocyte count}\left(\times 103\text{ cells}/\text{μL}\right)}$$

Following NHANES recommendations, we categorized race as Mexican American, Other Hispanic, non-Hispanic White, non-Hispanic Black, and other. Educational level was categorized as “less than high school,” “completed high school,” and “more than high school.” Marital status was categorized as married/cohabiting or living alone. Annual household income was categorized as <$20,000 or ≥$20,000. Physical activity was categorized as inactive, moderate, vigorous, both moderate and vigorous. Smoking behavior was characterized by having smoked at least 100 cigarettes during one’s lifetime. Drinking behavior was assessed through two 24-hour dietary recalls, and participants were classified as drinkers if they reported consuming alcohol during at least one of the recalls. Diabetes was determined by meeting at least one of the following criteria: glycated hemoglobin level ≥ 6.5%, fasting blood glucose ≥ 7.0 mmol/L, self-reported physician diagnosis of diabetes, or self-reported use of insulin or glucose-lowering medications.

### 2.5 Statistical analysis

Given the complex multistage probability sampling design of NHANES data, this study applied weighting using the sample weight calculation method recommended by NHANES, specifically the “WTSAF2YR” or “WTMEC2YR” weights variable, to enhance the reliability of the findings. Our objective was to explore sex differences in the relationship between AIP and α-klotho. Therefore, we compared baseline characteristics and performed multiple linear regressions separately for men and women. Furthermore, to address the severely skewed distribution of α-klotho data and reduce variability between variables, we log-transformed the a-klotho levels and expressed them as Ln(α-klotho) for analysis. In the baseline data, numerical variables with a normal or approximately normal distribution were presented as mean ± standard deviation; while those with a skewed distribution were reported as median (interquartile range). Categorical variables were expressed as frequency (percentage). Baseline characteristic comparisons based on AIP quartiles were conducted using a one-way analysis of variance or the Kruskal–Wallis test for continuous variables, and the chi-square test was used for categorical variables. Weighted multiple linear regression was used to test for independent associations between AIP and Ln(α-klotho) levels across three models. AIP was analyzed as both a continuous and categorical variable (quartiles). Model 1 did not adjust for any covariates. Model 2 adjusted for age, race, education level, marital status, and annual household income. Model 3 included adjustments for all covariates. In addition, to explore the potential nonlinear relationship between AIP and Ln(α-klotho) levels, smooth curve fitting and threshold effect analysis were conducted using EmpowerRCH software to determine the optimal break point. Two-piecewise regression models were applied to examine the relationship between AIP and Ln(α-klotho) levels on either side of the break point. Furthermore, subgroup analyses were performed to test the effects of different subgroups on the results and to explore potential heterogeneity among these subgroups.

## 3 Results

### 3.1 Population characteristics

The study included 6,649 participants (3,458 women and 3,191 men). Baseline characteristics of the study population, stratified by sex, are presented in **Table 1**. Given the close association between α-klotho and aging, we further stratified α-klotho levels by age groups. The results showed that α-klotho levels were 805.03 (664.03, 1002.90) pg/ml in the 40–59 age group and 786.73 (645.62, 959.74) pg/ml in the 60–79 age group, with a statistically significant difference (p = 0.001). The baseline characteristics of male and female participants, classified according to AIP quartiles (less than −0.25, −0.25 to −0.03, −0.03 to 0.19, and more than 0.19), are summarized in **Tables 2**, **3**, respectively. Among female participants, there were significant differences in age, race, education level, annual household income, hypertension, diabetes, liver disease, kidney failure, physical activity, and smoking and drinking behaviors (p < 0.05). SII increased significantly with increasing AIP (p < 0.001), while Ln(α-klotho) decreased significantly with increasing AIP (p < 0.001). Similarly, among male participants, significant differences were observed in age, race, hypertension, diabetes, and smoking and drinking behaviors (p < 0.05). However, no significant differences were observed for SII and Ln(α-klotho) (p > 0.05).

**Table 1:** Baseline characteristics of the study participants stratified by sex.

Variables		man	woman	P-value
N, %		3191	3458	
Age, years		55.00 [47.00, 64.00]	56.00 [48.00, 65.00]	0.079
Race, n, %				0.131
	Mexican American	477 ( 6.39)	561 ( 6.55)	
	Other Hispanic	373 ( 5.15)	425 ( 4.71)	
	Non-Hispanic White	1428 (73.04)	1496 (71.92)	
	Non-Hispanic Black	600 ( 8.87)	661 (10.27)	
	Other Race	313 ( 6.55)	316 ( 6.55)	
Education level, n, %				0.581
	Less than high school	921 (17.41)	959 (16.64)	
	Completed high school	692 (20.74)	736 (21.72)	
	More than high school	1577 (61.86)	1759 (61.64)	
Marital status, n, %				
	Married/Cohabiting	216208493.17 (76.75)	204370077.79 (65.25)	<0.001
	Living alone	860 (23.25)	1485 (34.75)	
annual household income, n, %				
	<$20000	30400337.28 (11.19)	46576075.85 (15.36)	<0.001
	≥$20000	2421 (88.81)	2466 (84.64)	
Hypertension, n, %		1479 (42.70)	1643 (43.12)	0.792
diabetes, n, %		860 (21.13)	801(17.11)	0.001
CHD, n, %		228 ( 6.46)	110 ( 2.59)	<0.001
Liver disease, n, %		182 ( 4.96)	173 ( 4.39)	0.465
Kidney failure, n, %		140 ( 2.84)	127 ( 2.98)	0.792
physical activity, n, %				
	Inactive	1754 (50.78)	2353 (64.39)	<0.001
	Moderate	665 (22.74)	740 (24.15)	
	Vigorous	580 (20.73)	284 ( 9.25)	
	Both moderate and vigorous	190 ( 5.75)	80 ( 2.21)	
Smoking behavior, n, %		1902 (55.99)	1358 (41.57)	<0.001
Drinking behavior, n, %		1056 (42.77)	701 (27.44)	<0.001
SII (median [IQR])		450.43 [326.85, 626.64]	471.11 [343.40, 663.06]	0.001
AIP (median [IQR])		0.02 [-0.18, 0.26]	-0.10 [-0.32, 0.11]	<0.001
α-klotho (median [IQR]), pg/ml		777.80 [646.50, 953.40]	816.81 [669.57, 1010.80]	<0.001

**Table 2:** Baseline characteristics of the female population.

Variables		Total	Q1	Q2	Q3	Q4	P-value
N, %		3458	1061 (32.96)	885 (25.17)	856 (23.68)	656 (18.18)	
Age, years		56 (48.00,65.00)	54.00 (46.00–63.00)	55.29 (48.00,64.00)	57.00 (50.00,67.00)	56.00 (48.00,65.00)	<0.001
Race, n, %							<0.001
	Mexican American	560 (6.55)	101 (4.0)	161 (7.6)	161 (7.8)	137 (8.0)	
	Other Hispanic	425 (4.71)	85 (3.0)	112 (5.1)	126 (5.7)	102 (5.9)	
	Non-Hispanic White	1496 (71.92)	451 (70.9)	359 (69.8)	362 (72.1)	324 (76.6)	
	Non-Hispanic Black	661 (10.27)	311 (15.1)	172 (10.6)	128 (8.1)	50 (3.9)	
	Other Race	316 (6.55)	113 (6.9)	81 (6.9)	79 (6.3)	43 (5.7)	
Education level, n, %							<0.001
	Less than high school	959 (16.64)	195 (9.6)	246 (17.0)	279 (20.5)	239 (23.9)	
	Completed high school	736(21.72)	183 (15.0)	197 (24.5)	211 (26.3)	145 (24.2)	
	More than high school	1759 (61.64)	683 (75.4)	442 (58.5)	364(53.2)	270 (51.9)	
Marital status, n, %							0.074
	Married/Cohabiting	1972 (65.25)	627 (69.6)	492 (62.5)	489 (63.6)	364 (63.4)	
	Living alone	1485 (34.75)	434 (30.4)	393 (37.5)	366 (36.4)	292 (36.6)	
annual household income, n, %							<0.001
	<$20000	822 (15.36)	193 (11.1)	198 (14.1)	219 (18.8)	212 (20.4)	
	≥$20000	2466 (84.64)	821 (88.9)	638 (85.9)	593 (81.2)	414 (79.6)	
Hypertension, n, %		1643 (43.12)	401 (32.3)	412 (43.4)	451 (48.0)	379 (56.0)	<0.001
diabetes, n, %		801 (17.11)	127 (7.6)	180 (13.8)	242 (20.7)	252 (34.2)	<0.001
CHD, n, %		110 (2.59)	21 (1.5)	25 (2.5)	31 (3.1)	33 (4.1)	0.055
Liver disease, n, %		173 (4.39)	38 (2.6)	43 (4.0)	39 (4.5)	53 (8.0)	0.002
Kidney failure, n, %		131 (2.91)	24 (1.9)	26 (2.4)	43 (3.9)	34 (4.6)	0.031
physical activity, n, %							0.478
	Inactive	2353 (64.39)	704 (63.8)	603 (61.7)	602 (67.3)	444 (65.5)	
	Moderate	740(24.15)	238 (24.4)	182(24.4)	187(24.2)	133 (23.2)	
	Vigorous	284 (9.25)	90 (8.9)	75(11.4)	57 (7.0)	62(9.7)	
	Both moderate and vigorous	80 (2.21)	29 (2.8)	25 (2.5)	10 (1.5)	16 (1.7)	
Smoking behavior, n, %		1358 (41.57)	376 (37.0)	329 (40.8)	325 (39.9)	328 (53.0)	<0.001
Drinking behavior, n, %		701 (27.44)	309 (38.7)	160 (22.2)	125 (24.0)	107 (18.6)	<0.001
SII		473.67 (344.14,669.75)	427.71 (319.57,615.59)	473.90 (351.60,630.69)	501.61 (365.43,717.45)	518.53 (369.66,732.56)	<0.001
Ln(α-klotho)		6.71 (6.51,6.92)	6.76 (6.53,6.99)	6.71 (6.53,6.93)	6.68 (6.49,6.88)	6.62 (6.44,6.82)	<0.001

CHD, coronary heart disease; SII, systemic immune-inflammatory index.

**Table 3:** Baseline characteristics of the male population.

Variables		Total	Q1	Q2	Q3	Q4	P-value
N (%)		3191	601 (18.21)	779 (24.83)	803 (24.08)	1007 (32.88)	
Age, years		55.00 (47.00,64.00)	59.00 (49.00,66.00)	55.00 (47.00,63.00)	56.00 (48.00,65.00)	53.00 (46.00,62.00)	<0.001
Race, n, %							<0.001
	Mexican American	477 (6.39)	53 (3.8)	94 (4.9)	138 (7.5)	192 (8.1)	
	Other Hispanic	373 (5.15)	54 (4.2)	85 (4.7)	109 (6.0)	125 (5.4)	
	Non-Hispanic White	1428 (73.06)	238 (69.9)	337 (74.3)	351 (70.7)	502 (75.5)	
	Non-Hispanic Black	599 (8.85)	201 (15.9)	183 (11.0)	121 (7.4)	94 (4.3)	
	Other Race	313 (6.55)	55 (6.1)	80 (5.0)	84 (8.3)	94 (6.7)	
Education level, n, %							0.744
	Less than high school	921 (17.41)	172 (18.0)	216 (16.8)	217 (16.8)	316 (18.0)	
	Completed high school	692 (20.74)	140 (22.6)	164 (19.8)	170 (18.9)	218 (21.8)	
	More than high school	1576 (61.85)	289 (59.4)	399 (63.4)	415 (64.3)	473 (60.3)	
Marital status, n, %							0.426
	Married/Cohabiting	2331 (76.77)	407 (73.6)	578 (78.5)	604 (77.9)	742 (76.4)	
	Living alone	859 (23.23)	194 (26.4)	201 (21.5)	199 (22.1)	265 (23.6)	
annual household income, n, %							0.512
	<$20000	610 (11.17)	133 (12.7)	136 (9.9)	144 (11.0)	197 (11.4)	
	≥$20000	2421 (88.83)	431 (87.3)	612 (90.1)	616 (89.0)	762 (88.6)	
hypertension, n, %		1479 (42.70)	262 (35.8)	343 (39.3)	360 (42.9)	514 (49.0)	0.002
diabetes, n, %		860 (21.13)	104 (14.3)	162 (14.3)	227 (22.5)	367 (29.0)	<0.001
CHD, n, %		228 (6.46)	42 (7.3)	45 (5.1)	56 (6.2)	85 (7.2)	0.474
Liver disease, n, %		182 (4.96)	35 (5.4)	37 (4.1)	48 (5.3)	62 (5.1)	0.788
Kidney failure, n, %		140 (2.84)	25 (3.2)	31 (2.2)	34 (3.2)	50 (2.8)	0.682
physical activity, n, %							0.587
	Inactive	1754 (50.79)	337 (53.1)	417 (48.4)	445 (53.1)	555 (49.7)	
	Moderate	664 (22.72)	124 (22.9)	153 (21.0)	179 (23.4)	208 (23.4)	
	Vigorous	580 (20.73)	108 (19.8)	150 (23.2)	139 (18.5)	183 (21.0)	
	Both moderate and vigorous	190 (5.75)	31 (4.2)	58 (7.4)	40 (5.0)	61 (5.9)	
Smoking behavior, n, %		1901 (55.98)	339 (50.9)	433 (50.9)	500 (61.5)	629 (58.6)	0.006
Drinking behavior, n, %		1056 (42.78)	254 (56.2)	272 (41.6)	249 (41.2)	281 (37.2)	<0.001
SII		451.71 (328.32,629.33)	407.02 (289.80,613.03)	451.69 (331.21,608.37)	449.14 (333.78,642.58)	464.06 (330.35,636.34)	0.051
Ln(α-klotho)		6.66 ± 0.01	6.64 ± 0.31	6.67 ± 0.30	6.66 ± 0.31	6.67 ± 0.32	0.668

CHD, coronary heart disease; SII, systemic immune-inflammatory index.

### 3.2 Multivariate linear regression model

Weighted multivariate linear regression were used to evaluate the correlation between AIP and Ln(α-klotho) in middle-aged and older women and men, respectively. In the women’s group, Models 1, 2, and 3 showed that AIP was significantly negatively correlated with Ln(α-klotho) (Model 1: β = -0.146, 95% confidence interval [CI] = -0.210, -0.082; Model 2: β = -0.127, 95% CI = -0.194, -0.060; Model 3: β = -0.129, 95% CI = -0.209, -0.048); the higher the AIP, the lower the Ln(α-klotho). After converting the continuous variables to categorical variables, in the Models 1 (Q3: β = -0.090, 95% CI = -0.138, -0.042; Q4: β = -0.133, 95% CI = -0.179, -0.087), 2 (Q3: β = -0.072, 95% CI = -0.123, -0.021; Q4: β = -0.118, 95% CI = -0.167, -0.070), and 3 (Q3: β = -0.071, 95% CI = -0.126, -0.016; Q4: β = -0.120, 95% CI = -0.178, -0.063) results, Ln(α-klotho) was significantly lower in participants with high AIP than in those with low AIP (Q1). The trend analysis was significant, and the adjusted trend was unchanged (p < 0.001) (**Table 4**). In the men’s group, the relationship between AIP and Ln(α-klotho) was not significant (p > 0.05) in any of the three models, irrespective of whether AIP was used as a continuous or categorical variable (**Table 5**).

**Table 4:** Association between the AIP and Ln(α-klotho) in woman.

	Model 1		Model 2		Model 3	
	β (95% CI)	P-value	β (95% CI)	P-value	β (95% CI)	P-value
Continuous	-0.146 (-0.210,-0.082)	<0.001	-0.127 (-0.194,-0.060)	<0.001	-0.129 (-0.209,-0.048)	0.002
Categories						
Q1	Ref		Ref		Ref	
Q2	-0.039 (-0.078,0.001)	0.054	-0.031 (-0.071,-0.008)	0.118	-0.037 (-0.080,0.005)	0.082
Q3	-0.090 (-0.138,-0.042)	<0.001	-0.072 (-0.123,-0.021)	0.007	-0.071 (-0.126,-0.016)	0.012
Q4	-0.133 (-0.179,-0.087)	<0.001	-0.118 (-0.167,-0.070)	<0.001	-0.120 (-0.178,-0.063)	<0.001
P for trend	-0.045 (-0.061,-0.029)	<0.001	-0.039 (-0.056,-0.022)	<0.001	-0.039 (-0.058,-0.020)	<0.001

Model 1: Univariate analysis.

Model 2: Adjusted for age, race, education level, marital status, annual household income.

Model 3: Adjusted for age, race, education level, marital status, annual household income, diabetes, hypertension, CHD, liver disease, kidney failure, physical activity, smoking behavior, drinking behavior, and SII.

CHD, coronary heart disease; CI, confidence interval; SII, systemic immune-inflammatory index.

**Table 5:** Association between the AIP and Ln(α-klotho) in man.

	Model1		Model2		Model3	
	β (95% CI)	P-value	β (95% CI)	P-value	β (95% CI)	P-value
Continuous	-0.024 (-0.020,0.069)	0.281	-0.015 (-0.033,0.062)	0.541	0.006 (-0.051,0.063)	0.830
Categories						
Q1	Ref		Ref		Ref	
Q2	0.022 (-0.028,0.071)	0.386	0.014 (-0.038,0.067)	0.590	-0.0002 (-0.051,0.051)	0.994
Q3	0.018 (-0.023,0.059)	0.389	0.011 (-0.031,0.053)	0.605	-0.001 (-0.046,0.047)	0.981
Q4	0.027 (-0.017,0.071)	0.221	0.018 (-0.029,0.065)	0.455	-0.004 (-0.045,0.053)	0.871
P for trend	0.007 (-0.006,0.020)	0.275	0.005 (-0.009,0.018)	0.510	0.001 (-0.014,0.017)	0.857

Model 1: Univariate analysis.

Model 2: Adjusted for age, race, education level, marital status, annual household income.

Model 3: Adjusted for age, race, education level, marital status, annual household income, diabetes, hypertension, CHD, liver disease, kidney failure, physical activity, smoking behavior, drinking behavior, and SII.

CHD, coronary heart disease; CI, confidence interval; SII, systemic immune-inflammatory index.

### 3.3 Smoothed curve fitting and threshold effect analysis

**Figure 2:**
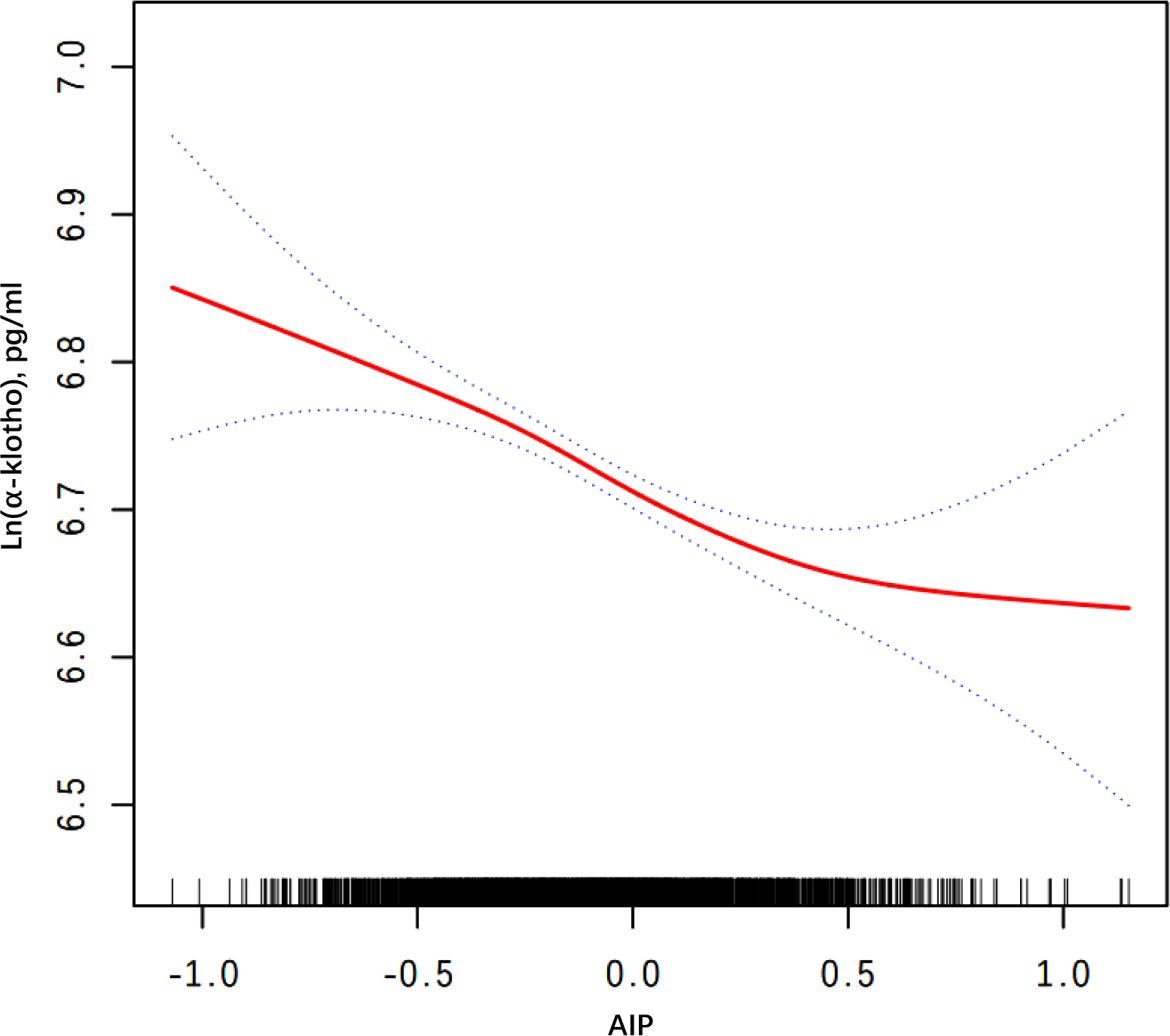
Smooth curve fitting for AIP and serum Ln(α-klotho) in woman. Adjusted for age, race, education level, marital status, annual household income, diabetes, hypertension, CHD, liver disease, kidney failure, physical activity, smoking behavior, drinking behavior, and SII. AIP, atherogenic index of plasma; CHD, coronary heart disease; SII, systemic immune-inflammatory index.

Smooth curve fitting and threshold effect analysis were used to explore the relationship between AIP and Ln(α-klotho) in female participants. The findings revealed a negative nonlinear relationship (**Figure 2**). Threshold effect analysis showed a significant difference in the association of AIP with α-klotho before and after the AIP break point of 0.434. At this break point, the value of Ln(α-klotho) was 6.618. Before the break point, there was a significant effect of AIP on Ln(α-klotho) (p < 0.001). However, after the break point, the relationship between AIP and Ln(α-klotho) was not significant (p = 0.051) (**Table 6**).

**Table 6:** Threshold effect analysis of the relationship between AIP and Ln(α-klotho) in woman.

Results of threshold effect analysis	Outcome	P-value
Model 1		
One-line effect	-0.127 (-0.167,-0.087)	<0.001
Model 2		
Break point (K)	0.434	
<K	-0.154 (-0.199,-0.110)	<0.001
>K	0.270 (-0.001,0.542)	0.051
Difference in effect before and after the breakpoint	0.425 (0.138,0.711)	0.004
α-klotho content at the folding point	6.618 (6.592,6.645)	
Logarithmic likelihood ratio test p-value		0.004

Adjusted for age, race, education level, marital status, annual household income, diabetes, hypertension, CHD, liver disease, kidney failure, physical activity, smoking behavior, drinking behavior, and SII.

CHD, coronary heart disease; SII, systemic immune-inflammatory index.

### 3.4 Subgroup analyses

**Figure 3:**
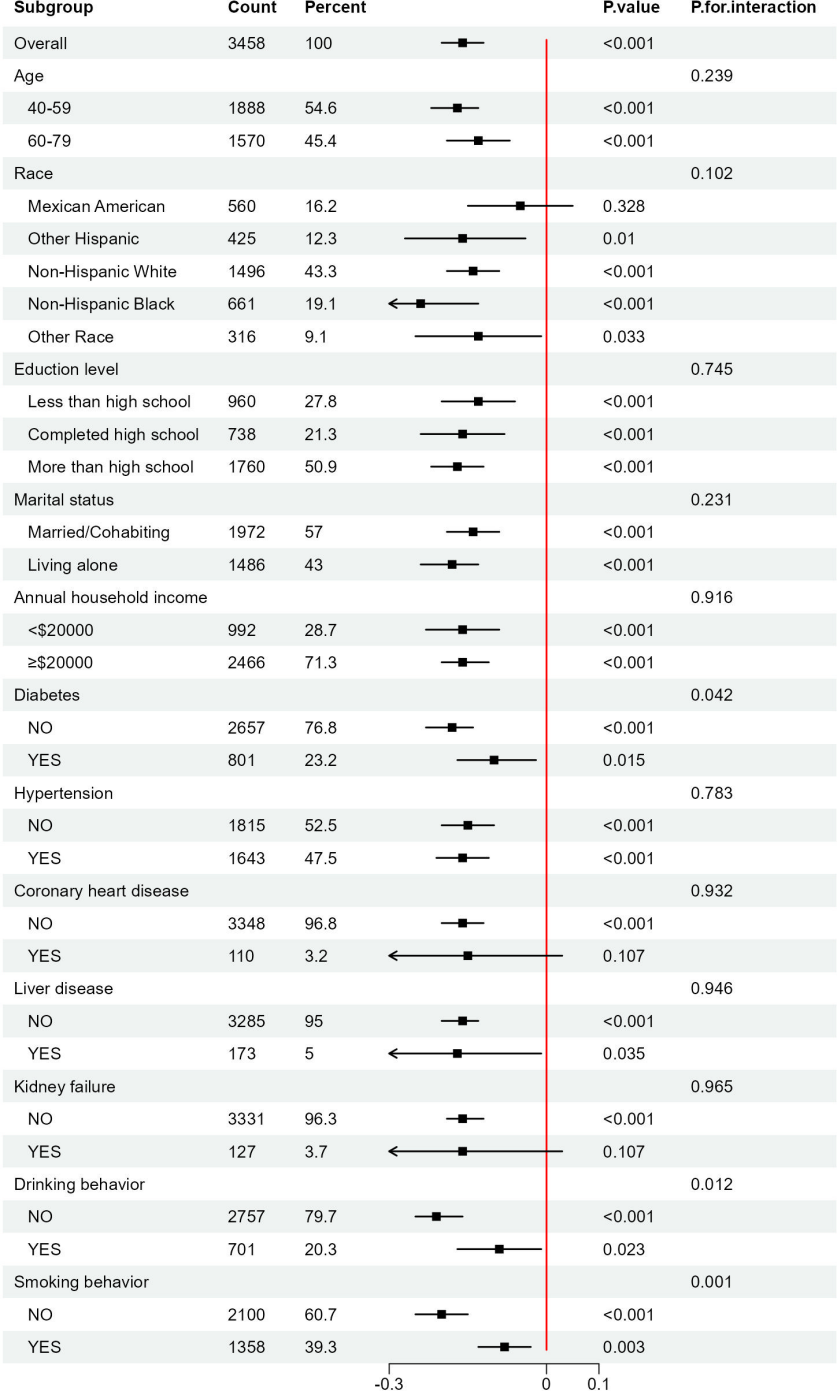
Subgroups analyses of the effect of AIP on Ln(α-klotho) in woman. AIP, atherogenic index of plasma.

Furthermore, to assess the consistency of the relationship between AIP and Ln(α-klotho) in different subgroups, we performed subgroup analyses and interaction tests in female participants. The findings indicated that AIP interacted with diabetes, smoking behavior, and drinking behavior (p for interaction < 0.05); however, the significant negative correlation between AIP and Ln(α-klotho) remained unchanged across subgroups (**Figure 3**). This indicates that the association between AIP and Ln(α-klotho) was consistent across various subgroups, suggesting our results’ reliability.

## 4 Discussion

Overall, this study included 6,649 participants, which comprised 3,458 women and 3,191 men. Univariate analysis showed that in female participants, Ln(α-klotho) decreased significantly with increasing AIP. However, in male participants, there was no significant difference in Ln(α-klotho) at different AIP levels. Furthermore, after adjusting for other covariates, weighted linear regression analyses of the three models consistently showed a negative correlation between AIP and Ln(α-klotho) in female participants, whereas the correlation was not significant in male participants. Subsequent smooth curve fitting analyses confirmed the negative nonlinear relationship between AIP and Ln(α-klotho) in female participants. However, threshold effect analyses confirmed the differential effect of AIP before and after the break point. Therefore, the results indicate a sex difference in the negative correlation between AIP and Ln(α-klotho); this negative correlation is significant in female participants, supporting its potential as a novel biomarker for evaluating serum α-klotho levels in women.

Current research has confirmed a decline in blood α-klotho levels with increasing age in healthy populations, with an even more pronounced reduction observed in individuals with kidney and endocrine/metabolic disorders (21). Moreover, age-related α-klotho deficiency is associated with medial calcification, intimal hyperplasia, endothelial dysfunction, arterial stiffness, hypertension, impaired angiogenesis, and vascular remodeling—hallmarks of early vascular aging (22). AIP serves as a crucial surrogate metabolic biomarker for atherosclerosis and cardiovascular disease (23). Currently, there is no direct evidence linking AIP to serum α-klotho; however, indirect evidence suggests such a connection. The two main components of AIP, TG and HDL-C, are involved in lipid metabolism. Previous studies have confirmed a potential link between lipid metabolism and aging. For instance, the aging process is associated with increased plasma lipoproteins and decreased plasma TG clearance due to decreased lipoprotein lipase activity (24). Furthermore, mean α-klotho levels were significantly lower in participants with elevated TG than in those with normal TG levels (25). In addition, klotho supplementation has been shown to reduce lipid accumulation and improved dyslipidemia in older adults (26). One study found that hypertriglyceridemia was independently associated with lower serum α-klotho levels; and this association was particularly pronounced in women (27). This finding aligns with our findings, where we observed a significant negative correlation between AIP, a novel indicator of lipid metabolism, and α-klotho levels, with this association present only in female participants.

Therefore, based on our findings, we suggest that the following mechanisms may mediate the association between AIP and serum α-klotho. First, α-klotho can inhibit the inflammatory response, potentially through the inhibition of nuclear factor kappa B and Wnt activity (28, 29). Liu et al. demonstrated in an *in vitro* model of chronic kidney disease that α-klotho reduces cellular inflammatory responses and improves cellular lipid metabolism (30). In addition, serum α-klotho can inhibit insulin signaling through the insulin-like growth factor receptor 1→ phosphatidylinositol 3 kinase (PI3K) → protein kinase B (Akt) → mammalian target of rapamycin complex 1 → peroxisome proliferator-activated receptor α pathway. This mechanism regulates energy metabolism in the liver and adipose tissue, promoting lipid oxidation and reducing lipogenesis (31). Finally, abnormal lipid metabolism can disrupt endoplasmic reticulum homeostasis, leading to the generation of large amounts of reactive oxygen species (32). However, α-klotho can mitigate oxidative stress induced by abnormal lipid metabolism by activating the PI3K/Akt/endothelial nitric oxide synthase pathway, downregulating lectin-like oxidized LDL receptor 1 expression, and upregulating oxidative scavengers (superoxide dismutase and nitric oxide) (33). Overall, the role of α-klotho in lipid metabolism appears complex and warrants further investigation.

Currently, the mechanisms underlying sex differences in the relationship between AIP and α-klotho remain unclear. One possible explanation is that sex hormones play distinct roles in regulating lipid dynamics, leading to sex differences in lipid profiles (34), These differences may contribute to the observed sex-specific association between AIP and α-klotho. In addition, the androgen receptor (AR) in the renal distal tubule is functionally associated with α-klotho. Androgens may bind to the AR, potentially influencing klotho gene expression (35), thereby contributing to differences in α-klotho levels between men and women. However, the exact role of this interaction in health outcomes is complex and requires further investigation. Since AIP can serve as an indicator for pharmacological interventions, further studies on the potential association between AIP and α-klotho levels, as well as sex differences in this association, are necessary.This is the first study to examine the relationship between AIP and α-klotho, revealing that higher AIP is associated with lower serum α-klotho levels. In addition to its originality, a significant strength of our study is the large sample size from NHANES and the reliability of its data sources, which minimizes bias.

Despite our promising results, this study has some limitations. First, due to its cross-sectional design, we could not establish a causal relationship between AIP and serum α-klotho protein levels. Therefore, further prospective cohort studies are necessary to investigate this causality in the future. Second, our study was based on a non-institutional US population aged 40–79, limiting the generalizability of our findings to other populations. Additional studies in diverse populations are needed to confirm these findings. Thirdly, although we controlled for several confounders, we could not account for all potential confounding factors. Some of these factors may act as mediators rather than true confounders in the observed associations. Therefore, our results should be interpreted with caution. Finally, while AIP shows promise as a biomarker, our study does not fully justify its use over LDL-C. Residual effects after LDL-C treatment may arise from genetic variability and apolipoprotein differences, which contribute to population heterogeneity. AIP alone cannot address this variability, highlighting the need for further research to compare the advantages and limitations of AIP and LDL-C in different populations.

## 5 Conclusions

Our study provides new evidence for sex differences in the association between AIP and serum α-klotho levels. AIP and serum α-klotho levels were negatively correlated in female participants but not significantly correlated in male participants. Further prospective studies in diverse populations are necessary to validate our findings and assess causality more thoroughly.

## Data Availability

Publicly available datasets were analyzed in this study. This data can be found here: www.cdc.gov/nchs/nhanes.htm.
